# Arterial and Venous Thrombotic Events in Urological Malignancy—A Real-World Cohort Study from a Tertiary Urology Referral Center

**DOI:** 10.3390/diagnostics16203298

**Published:** 2026-10-11

**Authors:** Cãtãlina-Antina Georgescu-Coriu, Ana-Maria Balahura, David-Andrei Anghel, Mihai Grigore, Diana-Ligia Pena, Adrian Coriu, Viorel Jinga, Camelia Nicolae, Adriana-Mihaela Ilieșiu

**Affiliations:** 1 Department of Cardiology, Faculty of Medicine, Carol Davila University of Medicine and Pharmacy, 050474 Bucharest, Romania; dr.coriucatalina@gmail.com (C.-A.G.-C.); andrei.david02@gmail.com (D.-A.A.); mihai.grigore@umfcd.ro (M.G.); dianaligiapena@gmail.com (D.-L.P.); viorel.jinga@umfcd.ro (V.J.); dr_camelia_nicolae@yahoo.com (C.N.); adriana.iliesiu@umfcd.ro (A.-M.I.); 2 Department of Cardiology, “Prof. Dr. Theodor Burghele” Clinical Hospital, 061344 Bucharest, Romania; 3 Department of Medical Oncology, “Prof. Dr. Theodor Burghele” Clinical Hospital, 061344 Bucharest, Romania; aacoriu@yahoo.com; 4 Department of Urology, “Prof. Dr. Theodor Burghele” Clinical Hospital, 061344 Bucharest, Romania

**Keywords:** cancer-associated thrombosis, urological malignancies, arterial thrombosis, venous thrombosis, cardiovascular disease, cancer stage, Khorana score, inflammatory indices

## Abstract

**Background/Objectives:** Cancer-associated thrombosis (CAT) increases morbidity and mortality in cancer patients. To describe CAT in urological malignancies and explore associated factors, timing, Khorana score performance and systemic inflammatory indices. **Methods**: This retrospective study included 1044 patients. Oncological, cardiovascular and laboratory data from medical records were compared by CAT status. Firth penalized logistic regression was used for sparse-event analyses. **Results:** Thirty-one patients (2.97%) developed CAT, including 17 arterial thrombotic events (ATE) and 14 venous thromboembolic events (VTE). Advanced stage was associated with CAT (adjusted odds ratio [aOR] per stage 1.60, 95% confidence interval [CI] 1.21–2.17; *p* < 0.001) in the primary and sensitivity analyses. In an exploratory analysis, cardiovascular disease was associated with ATE (odds ratio [OR] 4.58, 95% CI 1.67–15.16; Benjamini–Hochberg [BH]-adjusted q = 0.031), although the small number of ATE precluded a reliable subgroup conclusion. Median time from cancer diagnosis to CAT was 3 months. The Khorana score did not show useful VTE discrimination in the systemic chemotherapy subgroup (77 patients; four VTE; area under the receiver operating characteristic curve [AUC] 0.437, 95% CI 0.127–0.746). The neutrophil-to-lymphocyte ratio (NLR), platelet-to-lymphocyte ratio (PLR), systemic inflammation response index (SIRI), and systemic immune-inflammation index (SII) showed no useful discrimination (AUCs 0.487–0.513) or improvement of the clinical model (all BH-adjusted q = 0.963). **Conclusions**: Advanced stage was the most consistent correlate of CAT. Arterial and venous events occurred in similar proportions and were concentrated early after diagnosis. Exploratory analyses suggested a possible relationship between cardiovascular disease and ATE, whereas the Khorana findings were inconclusive and the inflammatory indices lacked discriminatory value. Larger prospective studies are needed to confirm these findings.

## 1. Introduction

Cancer-associated thrombosis (CAT), encompassing both venous thromboembolism (VTE) and arterial thromboembolism (ATE), is a major cause of morbidity and mortality in patients with active cancer, with VTE ranking as the second leading cause of death after cancer itself [1,2]. CAT risk is influenced by patient-related factors, tumor characteristics and stage, time since cancer diagnosis and anticancer therapies [2,3]. A nationwide study of over 158,000 patients with cancer reported ATE and VTE in 5.4% and 4.6% of patients, respectively [4]. Despite this substantial burden, evidence on CAT in urological malignancies remains limited, particularly for ATE. Several treatments used in urologic oncology, including gonadotropin-releasing hormone (GnRH) agonists, platinum-based chemotherapy, and poly (ADP-ribose) polymerase (PARP) inhibitors, have also been associated with an increased thromboembolic risk [3,5].

Venous thromboembolism (VTE), comprising deep vein thrombosis (DVT) and pulmonary embolism (PE), is 4–7 times more common in patients with cancer than in the general population [6]. It may occur at any stage and can be the first manifestation of an underlying malignancy [6]. The association between cancer and thrombosis was first described by Bouillard in 1823 and subsequently by Trousseau in 1844 [1]. The mechanisms of CAT are multifactorial, involving tumor-driven coagulation activation, impaired fibrinolysis, inflammatory and angiogenic mediators, and interactions among tumor cells, endothelial cells, leukocytes and platelets [1]. VTE increases perioperative complications, prolongs hospitalization, may delay anticancer therapy and leads to greater healthcare resource use [6]. Several models predict VTE risk, including the Khorana score for patients receiving chemotherapy [7]. However, its predictive accuracy in urological cancers appears limited [7,8]. Furthermore, ATE, including myocardial infarction and stroke, is associated with substantial morbidity and a poor prognosis [5]. Key mechanisms include endothelial injury, hypercoagulability and increased platelet reactivity [9].

The current study aims to describe the frequency of thrombotic events in a urological oncology cohort, compare patients with and without thrombosis, evaluate clinical and laboratory correlates, assess the discriminative performance of the Khorana score for VTE among patients receiving systemic chemotherapy and of inflammatory indices for overall thrombosis and describe the timing of thrombotic events after cancer diagnosis.

## 2. Materials and Methods

### 2.1. Study Design and Population

This retrospective observational cohort study was conducted at the Oncology Department including the Oncology Day Unit and the Outpatient Oncology Clinic, Prof. Dr. Theodor Burghele Clinical Hospital, a tertiary urology referral center in Bucharest, Romania. The study included adult (≥18 years) outpatients with pathologically confirmed urological cancers enrolled in a consecutive manner between January 2022 and November 2024. Cohort entry (enrollment) was defined as the first visit to our oncology department. The cancer diagnosis date was defined as the date of histopathological confirmation and could precede cohort entry. Individual follow-up extended from cohort entry to the last documented visit to our oncology department, with an administrative cutoff of 31 December 2024. Patients whose medical records were limited to the cancer diagnosis, without additional clinical information allowing patient characterization, were excluded. Primary urological malignancies were classified into five categories: renal, urothelial, prostate, testicular and penile cancers. Urothelial cancers included malignancies arising from the urinary bladder and upper urinary tract, including the ureter and renal pelvis.

The following clinical, oncological and laboratory data were collected from medical records: demographic data, cardiovascular disease, cardiovascular risk factors, cancer stage, histopathological characteristics, oncological treatment, anticoagulation status, laboratory parameters, the occurrence and type of thrombotic events and for patients who developed CAT, the time from cancer diagnosis to thrombosis.

CAT included both venous thrombosis and arterial thrombosis. Venous thrombotic events were defined as an intraluminal filling defect involving the pulmonary arterial circulation or any venous territory on contrast-enhanced computed tomography (CT) scans of the thorax, abdomen or pelvis, or as venous non-compressibility established by compression ultrasonography [10,11,12]. Intravascular tumor extension described as tumor thrombus in imaging reports was excluded from the thrombotic endpoint. Arterial thrombotic events were defined as objectively documented acute arterial thrombotic, thromboembolic or ischemic events, irrespective of vascular territory, diagnosed according to the clinical, laboratory and imaging criteria appropriate to each event [13,14,15]. The primary endpoint was the occurrence of CAT, comprising both VTE and ATE, after enrollment in our oncology department. Thrombotic events before enrollment were not counted as study outcomes. ATE and VTE were combined to describe the overall burden of thrombosis in this cohort. Given their different mechanisms and risk factors, the two components were also described separately; analyses by event type were considered exploratory.

Cardiovascular disease was considered present in patients with a documented history of heart failure, ischemic heart disease, cardiac arrhythmias, conduction disorders, valvular heart disease, systemic atherosclerotic disease or structural myocardial abnormalities, including cardiac hypertrophy or dilatation. Pre-existing cardiovascular disease was ascertained from medical records and patient history documented at the initial visit to our oncology department, before any thrombotic event counted as a study outcome.

Traditional cardiovascular risk factors included smoking, hypertension, diabetes mellitus, obesity and dyslipidemia. Autoimmune disease was recorded separately as an additional potential risk factor.

Cancer stage was obtained from medical records and recorded according to the documented Tumor-Node-Metastasis (TNM)-based stage classification [16].

Given the differences in tumor-specific histological grading systems, grading data were harmonized into 3 study-defined categories (low, intermediate and high) to facilitate statistical analyses. For prostate cancer, International Society of Urological Pathology (ISUP) Grade Group (GG) 1 was classified as low grade, GGs2–3 as intermediate grade and GG4–5 as high grade [17]. For renal cell carcinoma, World Health Organization (WHO)/ISUP grade 1 was classified as low grade, 2 as intermediate grade and 3–4 as high grade [18]. For urothelial carcinomas, tumors classified as low grade according to the WHO/ISUP grading system were assigned to the low-grade category, while those classified as high grade were assigned to the high-grade category [19]. For penile cancer, WHO/ISUP grade 1, grade 2 and grade 3 tumors were assigned to the low, intermediate and high-grade categories, while sarcomatoid tumors were included in the high-grade category [20,21]. Testicular tumors and chromophobe renal cell carcinomas for which the grading system was not applicable, were excluded from the tumor grade analysis [22,23].

Anticancer treatment exposure during the study observation period was recorded, including systemic chemotherapy, hormone therapy, immunotherapy, targeted therapy, radiotherapy, surgery and intravesical therapy (Bacillus Calmette–Guérin [BCG] immunotherapy or intravesical chemotherapy). These variables reflected documented treatment exposure at any time during the study observation period and in patients who developed CAT, exposure could have occurred before or after the thrombotic event. Recorded treatment exposures were coded as separate, non-mutually exclusive variables. Patients receiving treatments from different classes were counted in each applicable category. Patients who did not receive active anticancer treatment during the study period were classified as having no treatment. No antibody–drug conjugate treatment was recorded in the cohort. For patients who developed CAT, treatment status at the time of the thrombotic event was also recorded and analyzed descriptively. Treatment-at-event categories were mutually exclusive; no patient with thrombosis was receiving treatments from more than one category at the time of the event.

The Khorana score was assessed in patients with documented exposure to systemic chemotherapy, consistent with its intended clinical use. The score was calculated using cancer type (bladder + 1, testicular + 1), pre-chemotherapy platelet count (≥350 × 10^9^/L), hemoglobin level (<10 g/dL), prechemotherapy leucocyte count (>11 × 10^9^/L) and Body Mass Index BMI (≥35 kg/m^2^) [24]. The Khorana score was treated as an ordinal variable and a score ≥ 2 was considered indicative of increased VTE risk, in accordance with current American Society of Clinical Oncology (ASCO) recommendations [25].

The neutrophil-to-lymphocyte ratio (NLR), platelet-to-lymphocyte ratio (PLR), systemic inflammation response index (SIRI), and systemic immune-inflammation index (SII) were derived from laboratory parameters [26]. These indices were calculated from laboratory results available at the baseline oncology assessment, the reference point for patients with and without subsequent thrombosis. NLR was calculated as the absolute neutrophil count divided by the absolute lymphocyte count, while PLR was calculated as the platelet count divided by the absolute lymphocyte count [26]. SIRI was calculated as the product of the absolute neutrophil and monocyte counts divided by the absolute lymphocyte count, whereas SII was calculated as the product of the platelet and absolute neutrophil counts divided by the absolute lymphocyte count [26]. Not all the patients had available laboratory data; therefore, they were excluded from the statistical analyses of the systemic inflammatory indices (NLR, PLR, SIRI and SII).

### 2.2. Data Collection

Patient data were obtained from both paper-based and electronic hospital medical records. The collected data included demographics, tumor characteristics, oncological treatment, laboratory data, associated cardiovascular disease and risk factors, as well as characteristics of the thrombotic event, when present.

### 2.3. Statistical Analysis

The analysis was performed using R version 4.4.2 (“Pile of Leaves”). The analyses were exploratory and hypothesis-generating rather than confirmatory. Data quality and missingness were assessed before analysis. Only patients with complete laboratory data were included in analyses of laboratory-derived indices; missing values were not imputed. Continuous variables were summarized by median and interquartile range (IQR), appropriate for potentially skewed distributions. Categorical variables were summarized as counts and percentages. The Wilcoxon–Mann–Whitney test was used for two-group continuous comparisons. Fisher’s exact test was used for categorical variables because thrombotic events and some categories were sparse. Observation duration was calculated in months from enrollment to the last documented oncology visit within the study period, using registry entry and exit months, and summarized by thrombosis status and event type. The available dataset did not support formal time-to-event analyses incorporating individual follow-up duration, censoring, competing risks, or time-varying exposures. Accordingly, regression results are reported as odds ratios within the observed dataset and are not interpreted as incidence rates, hazards or causal effects.

Univariable and multivariable associations were estimated using Firth penalized logistic regression with profile-likelihood 95% confidence intervals (CIs). This method reduces small-sample and separation bias, which is important with only 31 events and very rare exposures, but does not overcome the limited statistical power associated with sparse events. Odds ratios (ORs), adjusted odds ratios (aORs), 95% CIs and *p*-values were reported. For exploratory families of multiple comparisons, Benjamini–Hochberg (BH) false-discovery-rate adjusted q-values were calculated. Interpretation emphasized effect estimates, uncertainty, and consistency across analyses.

The primary multivariable model included age per 10-year increase, cardiovascular disease, number of cardiovascular risk factors and diagnostic cancer stage. Collinearity among the model covariates was assessed using variance inflation factors (VIFs). All values were below 1.3: 1.20 for cardiovascular disease, 1.15 for the number of cardiovascular risk factors, 1.06 for age, and 1.02 for cancer stage, suggesting little multicollinearity. Cancer stage was modeled as an ordinal variable (0–IV) per one-level increase, assuming a linear effect across stage levels on the log-odds of thrombosis. This assumption was not formally tested given the small number of events. Apparent discrimination was assessed using the area under the receiver operating characteristic curve (AUC) in the development sample. Given the biological and therapeutic heterogeneity of the included malignancies, pooled estimates were interpreted as associations within the overall urological cancer cohort rather than as cancer-specific effect estimates. To assess whether cancer-type heterogeneity materially influenced the primary clinical associations, an exploratory sensitivity model additionally adjusted for cancer type. Prostate, renal, and urothelial cancers were retained as separate categories, while penile and testicular cancers were combined as “Other” because of their small numbers. Cancer type was included as an adjustment variable to assess the stability of the primary clinical associations rather than to estimate cancer-specific risk. A second sensitivity analysis compared stage IV with stages 0–III, with and without adjustment for cancer type. In a further exploratory sensitivity analysis, the primary model was refitted with registry-derived observation duration included as an additional covariate.

Exploratory univariable treatment analyses were based on documented treatment exposure at any time during the study observation period. For patients who developed thrombosis, recorded exposure could therefore have occurred before or after the thrombotic event and was not considered a time-ordered exposure. These analyses were interpreted as associations between documented treatment exposure and thrombosis status rather than as estimates of treatment-related thrombotic risk. Because treatment allocation was non-random and temporal ordering relative to thrombosis was not established, no multivariable treatment-effect models were performed. Treatment status at the time of thrombosis was analyzed separately and descriptively among event cases only. The treatment analyses evaluated nine recorded exposure categories, listed in Supplementary Table S4. Benjamini–Hochberg correction was applied to the available *p*-values from the univariable Firth regression comparisons. The *p*-values in Supplementary Table S3 are from descriptive Fisher’s exact tests and were not used for this correction.

The Khorana score analysis was restricted to patients with documented systemic chemotherapy exposure, with VTE as the endpoint. The score was compared between VTE and non-VTE groups using the Wilcoxon–Mann–Whitney test, evaluated per point using Firth logistic regression, and assessed by receiver operating characteristic (ROC) analysis with the AUC and 95% CI. At the prespecified threshold of ≥2, sensitivity, specificity, positive predictive value (PPV), and negative predictive value (NPV) were calculated with exact binomial 95% CIs.

NLR, PLR, SIRI, and SII were evaluated in the common complete-laboratory sample. Individual discrimination was assessed using ROC analysis with AUC and 95% CIs. For adjusted and incremental discrimination, the primary clinical model was refitted in the common complete-case population, and each inflammatory index was added separately. Adjusted associations were reported as ORs with 95%Cis. Paired DeLong tests compared each expanded model with the clinical model, with BH adjustment applied across the four comparisons.

Time from cancer diagnosis to thrombosis was calculated in months from histopathological confirmation to the thrombotic event and summarized using the median, IQR, range and clinically interpretable categories. These analyses were descriptive and were not interpreted as estimates of time-dependent risk.

## 3. Results

### 3.1. Cohort Characteristics and Event Burden

Of the 1166 patients with urological cancers identified in the oncology registry, 122 were excluded because their records lacked clinical information beyond the cancer diagnosis. The remaining 1044 patients had histologically confirmed urological malignancies, with a median age of 70 years (interquartile range [IQR] 64–75); 892 (85.4%) were male. The cohort comprised 440 prostate (42.1%), 110 renal (10.5%), 479 urothelial cancer (45.9%), one penile (0.1%) and 14 testicular (1.3%) cancers. Baseline demographic and oncological characteristics are summarized in Table 1. Thrombosis occurred in 31 patients (2.97%; 95% CI 2.03–4.19%), of whom 17 (54.8%) had arterial and 14 (45.2%) venous events, representing 1.63% and 1.34% of the overall cohort, respectively. The characteristics of the 31 thrombotic events, including event type, anatomical territory, cancer type, cancer stage and symptomatic or incidental presentation, are summarized in Table 2. Registry-derived median observation duration was 8 months (IQR 2–16) for patients without thrombosis, 11 months (IQR 3–20) for those with arterial events and 8.5 months (IQR 4.5–20.8) for those with venous events.

Median age was 73 years in patients with thrombosis and 69 years in those without thrombosis (*p* = 0.200). By cancer type, thrombosis occurred in 11/440 patients with prostate cancer (2.50%), 6/110 with renal cancer (5.45%), 13/479 with urothelial cancer (2.71%), 1/1 with penile cancer, and 0/14 with testicular cancer. The overall distribution of cancer types differed according to thrombosis status (Fisher’s exact *p* = 0.029); however, this comparison was limited by sparse categories, particularly penile cancer, which was represented by a single patient who developed thrombosis. Therefore, the finding should not be interpreted as evidence of cancer-specific differences in thrombotic risk.

Stage IV disease was present in 14/31 (45.2%) patients with thrombosis compared with 207/1013 (20.4%) patients without thrombosis. By event type, stage IV disease was present in 8/17 patients with ATE (47.1%) and 6/14 with VTE (42.9%), compared with 207/1013 patients without thrombosis (20.4%). The full stage distribution by event type is shown in Supplementary Table S1. Histopathological grade did not differ between groups (*p* = 0.5), with high-grade tumors present in 64.5% of patients with thrombosis and 53.1% of those without thrombosis.

### 3.2. Cardiovascular Profile

Cardiovascular disease was present in 19/31 (61.3%) patients with thrombosis compared with 401/1013 (39.6%) without thrombosis (Fisher’s exact *p* = 0.024). In exploratory univariable Firth regression, cardiovascular disease was associated with higher odds of thrombosis (OR 2.38, 95% CI 1.17–5.02; *p* = 0.017), although the BH-adjusted q-value was 0.192. Subtype analyses included only 17 arterial and 14 venous events and were therefore considered exploratory. In the exploratory comparison of ATE versus no thrombosis, cardiovascular disease was associated with higher odds of ATE (OR 4.58, 95% CI 1.67–15.16; BH q = 0.031). Although this association remained significant after correction for multiple testing, it should be interpreted cautiously given the small number of arterial events.

**Figure 1:**
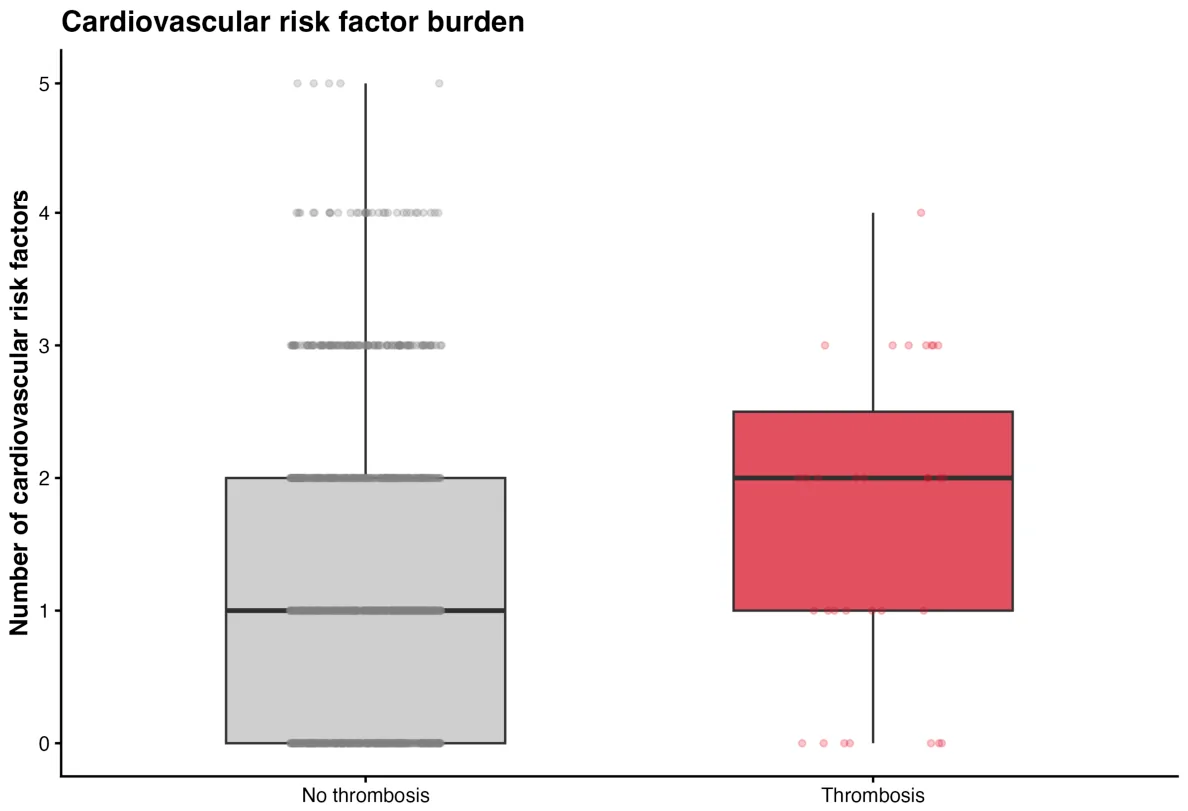
Cardiovascular risk-factor burden according to thrombosis status—Patients with thrombosis had a higher median number of cardiovascular risk factors than those without thrombosis (2 [IQR 1–3] vs. 1 [IQR 0–2]), although the difference was not statistically significant (*p* = 0.068).

The burden of cardiovascular risk factors was numerically higher among patients with thrombosis than among those without thrombosis (median 2 [IQR 1–3] vs. 1 [IQR 0–2], *p* = 0.068) (Figure 1). In descriptive subtype analyses, patients with ATE had a higher median number of cardiovascular risk factors than those with VTE (2 vs. 1, *p* = 0.035); given the small number of subtype events, this finding should be interpreted cautiously. In the exploratory comparison of ATE versus no thrombosis, each additional cardiovascular risk factor was associated with higher odds of ATE (OR 1.65, 95% CI 1.13–2.40; *p* = 0.010), although this association did not remain statistically significant after BH correction (q = 0.079).

Anticoagulant treatment was recorded in 65 patients (6.2%) overall, including 62 (6.1%) patients without thrombosis and three (9.7%) with thrombosis (*p* = 0.400). Anticoagulation status reflected treatment before the thrombotic event; however, information on the indication for anticoagulation, including whether it was prescribed for therapeutic or prophylactic purposes, was not available.

### 3.3. Primary Multivariable Model and Sensitivity Analyses

In the primary Firth model, each one-level increase in diagnostic stage was associated with higher odds of thrombosis (aOR 1.60, 95% CI 1.21–2.17; *p* < 0.001), whereas age, cardiovascular disease, and the number of cardiovascular risk factors were not statistically significant (Table 3). The apparent AUC of the primary model was 0.727 (95% CI 0.652–0.803), representing development-sample performance rather than validated prediction. The association between more advanced stage and thrombosis was preserved after additional adjustment for cancer type (aOR per one-level increase 1.89, 95% CI 1.38–2.61; *p* < 0.001, Supplementary Table S2). When stage IV was contrasted with stages 0–III, stage IV was associated with higher odds of thrombosis both without (aOR 3.27, 95% CI 1.57–6.74; *p* = 0.002) and with additional adjustment for cancer type (aOR 4.18, 95% CI 1.87–9.29; *p* < 0.001). In a further exploratory model that included registry-derived observation duration, the estimates for cardiovascular disease (aOR 2.11 in both models) and diagnostic stage (aOR 1.57 versus 1.60 per stage level in the primary model) were similar.

### 3.4. Laboratory Variables and Inflammatory Indices

None of the inflammatory indices showed useful individual discrimination for thrombosis, with AUCs ranging from 0.487 for SIRI to 0.513 for PLR (Table 4). In adjusted complete-case models, none of the four indices was associated with thrombosis, while the association with diagnostic stage remained stable across models (aOR approximately 1.80–1.83 per one-level increase).

The clinical model AUC in the common sample was 0.757 (95% CI 0.683–0.831). Adding NLR, PLR, SIRI, or SII separately resulted in minimal changes in discrimination (maximum ΔAUC approximately 0.003), with no significant improvement compared with the clinical model alone (all BH-adjusted q = 0.963) (Table 4).

### 3.5. Khorana Score for Venous Thrombosis

**Figure 2:**
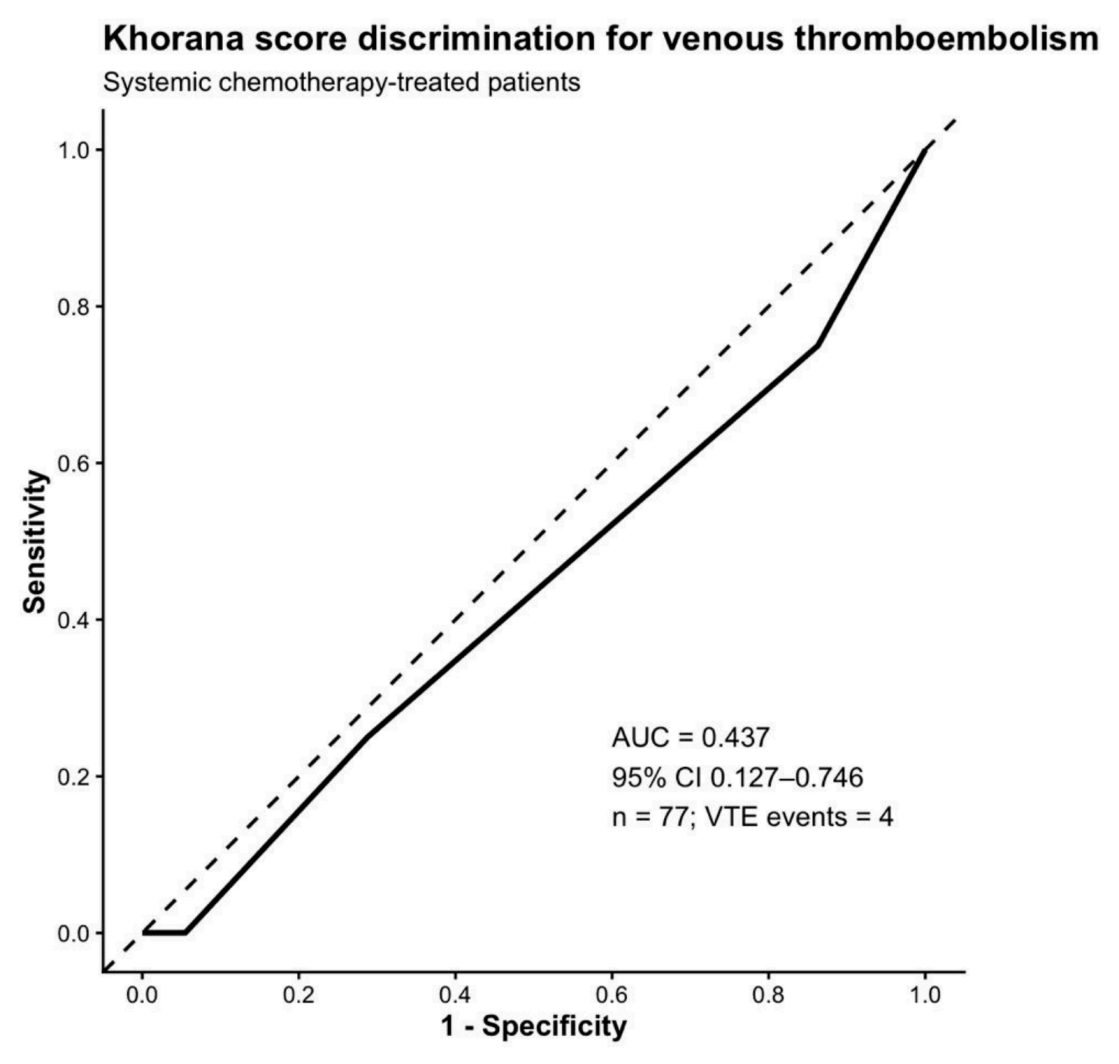
Discrimination of the Khorana score for venous thromboembolism among systemic chemotherapy-treated patients—ROC curve for the Khorana score among 77 evaluable patients receiving systemic chemotherapy, including four VTE. The AUC was 0.437 (95% CI, 0.127–0.746). The diagonal dashed line represents discrimination equivalent to chance (AUC = 0.50). Given the small number of VTE events, the estimate is highly imprecise and should be interpreted as exploratory.

Of the 79 patients with recorded systemic chemotherapy exposure, two had no pre-chemotherapy blood count results available and could not be scored. The Khorana score was therefore evaluated in 77 patients, among whom four VTE events occurred. Median Khorana score was 1 in both VTE and non-VTE groups (Wilcoxon *p* = 0.644). The per-point Firth OR was 0.79 (95% CI 0.164–2.44; *p* = 0.731). The AUC was 0.437 (95% CI 0.127–0.746), providing no evidence of useful discrimination in this dataset; the wide interval reflects the extremely small number of VTE events and precludes a definitive conclusion about performance (Figure 2).

At a Khorana score ≥ 2, there was one true positive (TP), 21 false positives (FP), 52 true negatives (TN), and three false negatives (FN). Sensitivity was 25.0% (95% CI 0.6–80.6%), specificity 71.2% (95% CI 59.4–81.2%), PPV 4.5% (95% CI 0.1–22.8%), and NPV 94.5% (95% CI 84.9–98.9%).

### 3.6. Cancer Treatment and Thrombosis

Recorded systemic chemotherapy exposure was present in 9/31 (29.0%) thrombosis cases versus 70/1013 (6.9%) non-cases; the exploratory univariable Firth OR was 5.65 (95% CI 2.44–12.20; *p* < 0.001; BH q = 0.001). Recorded immunotherapy exposure was present in 4/31 (12.9%) thrombosis cases versus 21/1013 (2.1%) non-cases; the exploratory univariable Firth OR was 7.55 (95% CI 2.26–20.73; *p* = 0.002; BH q = 0.011; Supplementary Tables S3 and S4). These estimates should not be interpreted as treatment effects because treatment allocation was non-random, cancer type and treatment were strongly related and timing of recorded exposure relative to thrombosis was not established.

**Figure 3:**
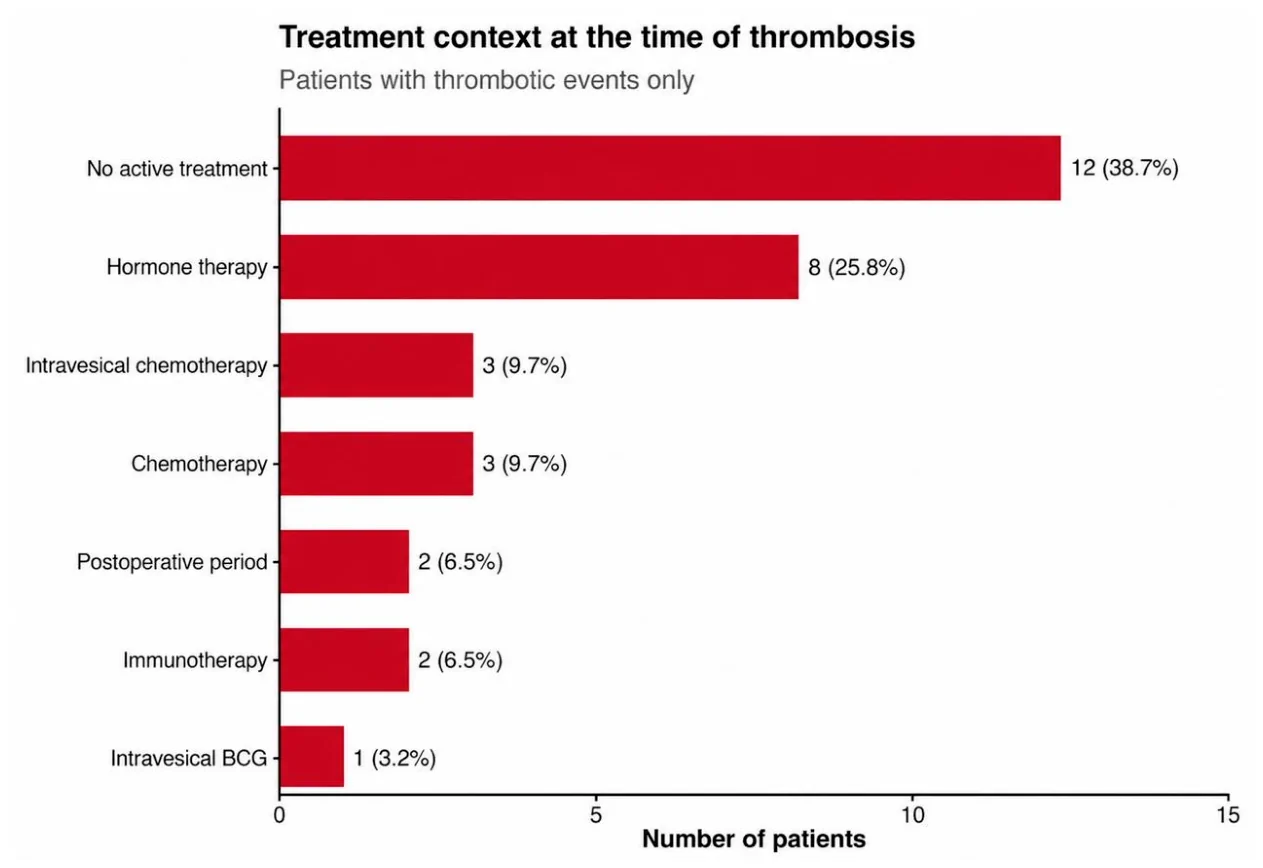
Treatment context at the time of thrombosis—Categories are mutually exclusive, with each of the 31 patients counted once. No active-treatment category was applicable in 38.7% of cases; hormone therapy was the most frequent treatment category at the event. Note: Percentages may not sum to 100% due to rounding.

The distinction between recorded exposure and treatment-at-event was material. Among the 31 thrombosis cases, nine had recorded systemic chemotherapy exposure but only three were receiving chemotherapy at the event; hormone therapy was recorded in 10 but active at the event in eight; intravesical chemotherapy was recorded in four but active at the event in three; and immunotherapy was recorded in four but active at the event in two. At the thrombotic event, 12/31 (38.7%) had no applicable active-treatment category, 8/31 (25.8%) were receiving hormone therapy, 3/31 (9.7%) systemic chemotherapy, 3/31 (9.7%) intravesical chemotherapy, 2/31 (6.5%) immunotherapy, 2/31 (6.5%) were postoperative, and 1/31 (3.2%) was receiving intravesical BCG (Figure 3).

### 3.7. Timing Context of Thrombosis

**Figure 4:**
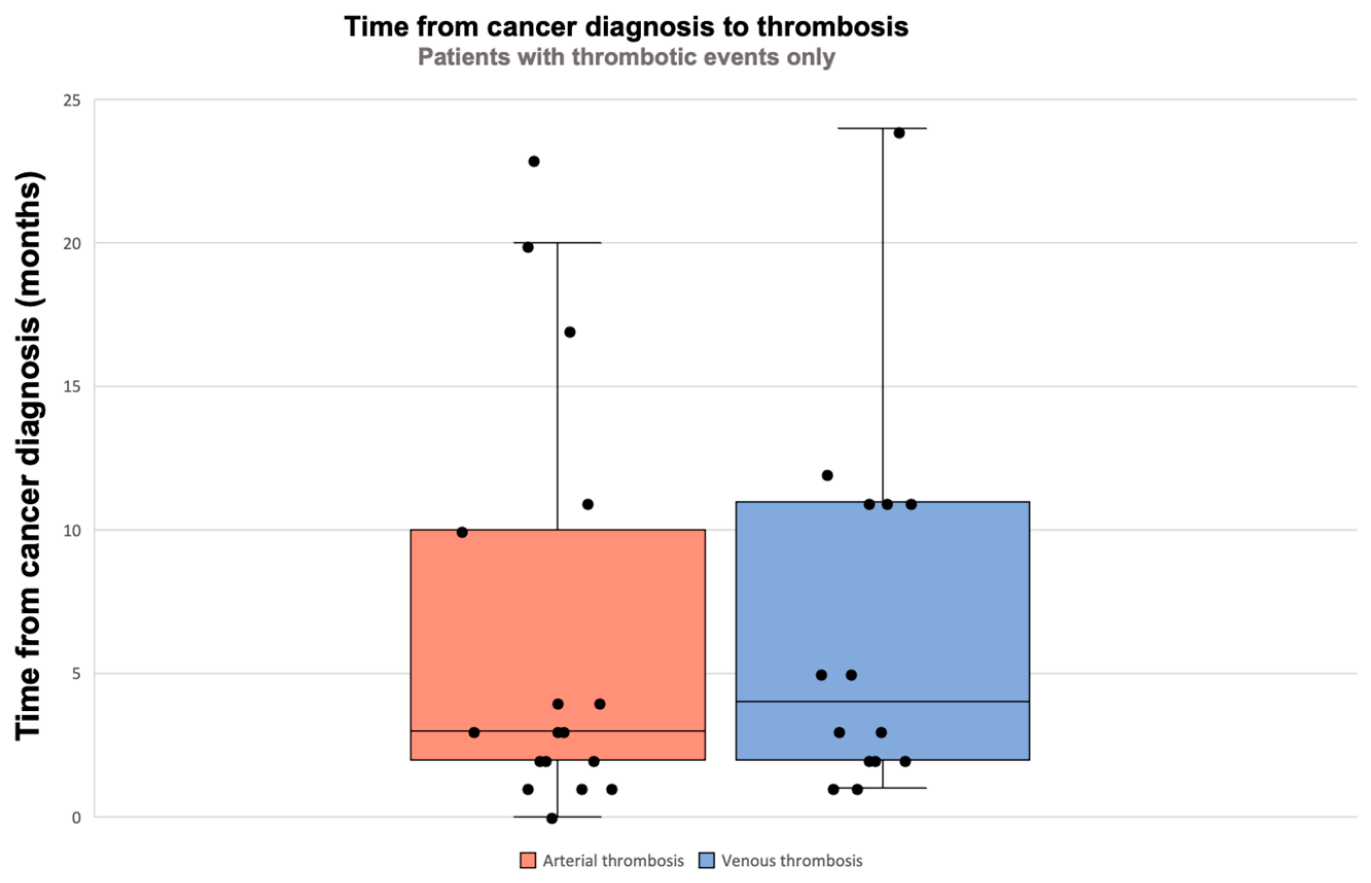
Time from cancer diagnosis to thrombosis according to thrombotic event type—Distribution of arterial and venous thrombotic events according to time from cancer diagnosis. Event-type stratification is presented descriptively.

Median time from cancer diagnosis to thrombosis was 3 months (IQR 2–11; range 0–24) (Figure 4). Seventeen events (54.8%) occurred within three months of cancer diagnosis, four (12.9%) at 4–6 months, six (19.4%) at 7–12 months and four (12.9%) after 12 months. These observations are descriptive and do not estimate a time-dependent hazard.

## 4. Discussion

Our study provides insight into the overall thrombotic burden and associated clinical factors in patients with urological malignancies by considering a broad spectrum of both venous and arterial events. This is particularly relevant given the limited literature on CAT encompassing both event types, as most previous studies have focused primarily on DVT and PE.

CAT occurred in 2.97% of patients in our cohort, with arterial events accounting for slightly more than half of all thrombotic events. Direct comparison with previous studies is difficult, as data combining VTE and ATE in urological cancers remain limited, with most studies focusing on VTE. Nevertheless, a large population-based study found that among the cancer types evaluated, urinary tract malignancies had the highest relative risk of ATE compared with individuals without [4]. A recent meta-analysis of 230,660 patients with urogenital cancers reported a pooled VTE proportion of approximately 3% [27]. In a separate cohort of 354 patients with advanced urinary tract cancer receiving first-line chemotherapy, ATE occurred in 3.4% [28]. Differences in study populations, disease stage, treatment exposure and outcome definitions should be considered when comparing these findings.

The median age of patients with CAT was 73 years, similar to the 69–71 years reported in studies of VTE in urological cancers [29,30]. However, age was not independently associated with thrombosis in our primary multivariable model.

In our study, cancer-type distribution differed between patients with and without CAT (*p* = 0.029). However, this comparison was limited by sparse categories, particularly the inclusion of a single patient with penile cancer who developed thrombosis, and should not be interpreted as evidence of cancer-specific differences in thrombotic risk. A recent meta-analysis reported substantial variability in VTE rates across urogenital cancers [27]. Evidence in this setting largely concerns VTE while arterial events remain less studied [7,31].

A recent meta-analysis across different cancer types showed that VTE risk increased with advancing cancer stage, while an analysis of the linked Surveillance, Epidemiology, and End Results–Medicare database similarly found a progressive increase in ATE with higher cancer stage [27,32]. These findings are consistent with our results, in which each one-level increase in cancer stage was associated with higher odds of CAT in the primary multivariable model. This association remained present after adjustment for cancer type and in the sensitivity analysis comparing stage IV with stages 0–III.

Higher histopathological tumor grade has been associated with an increased risk of VTE, while data regarding its relationship with ATE remain scarce [33]. Although in our cohort high-grade tumors were more frequent among patients with CAT, this difference was not statistically significant and should be interpreted with caution.

Cardiovascular disease was associated with overall CAT in the univariable analysis, but this association did not remain significant after correction for multiple testing and was not statistically significant in the primary multivariable model. In the exploratory analysis of ATE versus no thrombosis, cardiovascular disease remained associated with higher odds of ATE after correction for multiple testing. However, this finding was based on only 17 arterial events and should be interpreted cautiously. This finding is consistent with two recent large cohort studies, including more than 17,000 and 1.7 million patients with various types of cancers, which identified pre-existing cardiovascular disease as a predictor of ATE [34,35]. Literature regarding association between cardiovascular disease and VTE remains limited.

In descriptive subtype analyses, cardiovascular risk-factor burden was higher among patients with ATE than among those with VTE. However, with only 17 arterial and 14 venous events, this comparison was underpowered and cannot establish a difference between the two event types. In the exploratory comparison of ATE with no thrombosis, each additional cardiovascular risk factor was associated with higher odds of ATE, although this association did not remain statistically significant after correction for multiple testing. These observations are consistent with recent large cohort studies linking traditional cardiovascular risk factors to ATE in patients with cancer, but require confirmation in larger cohorts [34,35].

Pre-event anticoagulant use was numerically more frequent among patients with CAT than among those without thrombosis, but the difference was not statistically significant. Interpretation is limited by the fact that only three patients with CAT were receiving anticoagulant treatment and by the lack of information on treatment indications, including whether anticoagulation was prescribed for prophylactic or therapeutic purposes. Therefore, these data cannot be used to assess the effectiveness of anticoagulation in preventing thrombosis. Although anticoagulation can prevent VTE in selected patients with cancer, its role in ATE prevention remains less clear [36,37].

NLR, PLR, SII, and SIRI did not provide useful individual discrimination for CAT in our cohort, with AUC values close to 0.5. Moreover, adding each index separately to the clinical model resulted in minimal changes in AUC and did not significantly improve discrimination. Similarly, a prospective study found no association between PLR and VTE or ATE, while higher NLR showed only a non-significant trend for VTE [38]. Although these inflammatory indices have been associated with various malignancies and may reflect the systemic inflammatory component of cancer, they did not demonstrate useful discrimination for CAT in our cohort [39,40,41,42]. However, the limited number of thrombotic events in the complete-case analysis precludes definitive conclusions.

Among patients receiving systemic chemotherapy, the Khorana score did not show useful discrimination for VTE (AUC 0.437, 95% CI 0.127–0.746). However, only four VTE events occurred in this subgroup, leading to a wide confidence interval and preventing firm conclusions about the score’s performance. Similar findings have been reported in previous studies of patients with urological cancers [7,8].

Recorded exposure to systemic chemotherapy was more frequent among patients with CAT and was associated with higher odds of thrombosis in the exploratory univariable analysis. However, of the nine CAT patients with recorded systemic chemotherapy exposure, three were receiving chemotherapy at the time of the event. The observed association should therefore not be interpreted as a treatment effect, as treatment allocation was non-random, chemotherapy exposure was closely related to cancer type and stage, and the timing of exposure relative to thrombosis could not be established. Platinum agents such as cisplatin and carboplatin have been associated with an increased thrombotic risk, but our data cannot determine whether the observed association reflects chemotherapy, differences in disease characteristics or both [3,43].

Recorded immunotherapy exposure was also more frequent among patients with CAT and was associated with higher odds of thrombosis in the exploratory univariable analysis. However, of the four CAT patients with recorded immunotherapy exposure, two were receiving immunotherapy at the time of the event. Previous studies have shown conflicting results regarding the association between immunotherapy and CAT. A 279-patient cohort with metastatic urothelial carcinoma undergoing immunotherapy concluded that immune checkpoint inhibitors (ICI) are associated with a high thromboembolism risk [44]. In contrast, a retrospective multi-center study, with 407 enrolled patients affected by renal or bladder cancer, undergoing immunotherapy suggested that there is no association between ICI and VTE/ATE in these patients [45]. Because immunotherapy is more commonly used in advanced urological malignancies, our study cannot determine whether the observed association reflects immunotherapy exposure, more advanced disease or both [46].

Previous studies, including a 2018 systematic review and meta-analysis, have linked androgen deprivation therapy (ADT) to an increased risk of thromboembolic events, particularly DVT and PE [3,47]. In our cohort, recorded hormone therapy exposure was not significantly associated with CAT. Of the 10 CAT patients with recorded hormone therapy exposure, eight were receiving hormone therapy at the time of thrombosis. However, the absence of a significant association should not be interpreted as evidence that hormone therapy has no effect, given the limited number of events and the observational design of the study.

The thrombotic risk of intravesical therapy remains poorly characterized, as previous studies have mainly focused on other bladder cancer treatments [48]. In our cohort, recorded exposure to intravesical therapy was not significantly associated with CAT. Among the four CAT patients with recorded intravesical chemotherapy exposure, three were receiving intravesical chemotherapy at the time of thrombosis. Separately, one patient with CAT was receiving intravesical BCG when thrombosis occurred. Given the small number of events and limited available evidence, no firm conclusions can be drawn regarding the thrombotic risk of intravesical therapy.

At the time of thrombosis, no applicable active-treatment category was identified in 12 of the 31 patients with CAT. This category should not be interpreted as indicating that these patients had never received anticancer treatment. It may reflect different clinical settings, including newly diagnosed patients undergoing diagnostic work-up, patients under active surveillance, advanced disease with poor fitness for treatment or treatment refusal. Cancer-related prothrombotic mechanisms may contribute to CAT independently of anticancer treatment [9]. However, this descriptive finding cannot establish the relative contributions of the malignancy and its treatment.

Only two of the 31 CAT events occurred in a postoperative context. Given the small number of events and the outpatient setting of our cohort, these findings cannot be used to estimate postoperative thrombotic risk. Perioperative thromboprophylaxis may have influenced the number of events, but its role could not be assessed in our study [49]. Previous studies have reported VTE rates of 0.2–15.9% after urological cancer surgery, with the highest risk following cystectomy, open prostatectomy with extended pelvic lymph node dissection and high-risk renal procedures [50].

The median time from cancer diagnosis to thrombosis was three months, with 58.4% of events occurring within the first three months. This early concentration of events is consistent with previous studies [51,52]. A recent 2025 meta-analysis across different cancer types reported an increased risk of both VTE and ATE early in the course of cancer, which declined over time and eventually became non-significant [51]. The occurrence of most events early after diagnosis may indicate a clinically vulnerable period, although our descriptive analysis does not estimate time-dependent hazard or account for follow-up duration.

This research, however, is subject to several limitations. First, the retrospective, single-center design may limit generalizability. Despite the relatively large cohort, the small number of events (31 overall; 17 arterial and 14 venous) limited statistical power and precluded reliable comparisons across thrombotic subtypes, cancer types and treatment groups. The primary model’s AUC was calculated in the development sample and may therefore be optimistic. Bootstrap internal validation was not performed, with only 31 events, an optimism-corrected estimate would also be imprecise. The inclusion of five urological cancer types with different biological characteristics, stages and treatments also introduced heterogeneity that could not be fully addressed by adjustment for cancer type. Therefore, the findings should be considered hypothesis-generating rather than confirmatory.

The observational design remains susceptible to residual confounding. Although treatment at the time of thrombosis was recorded, overall treatment exposure was assessed across the observational period and did not necessarily precede thrombosis. Treatment-related findings should therefore be interpreted cautiously. Exact dates of last follow-up were not available; observation duration was estimated from registry entry and exit months. Because logistic regression did not account for differences in the time available to observe thrombosis, unequal observation periods may have influenced the estimated associations. As time-to-event methods were not used, the observed early clustering of events after cancer diagnosis remains descriptive.

Third, data on several factors potentially contributing to thromboembolic risk were unavailable, including laboratory biomarkers (e.g., D-dimer, P-selectin), administration of erythropoiesis-stimulating agents (ESA) and presence of venous catheters, limiting a more comprehensive assessment of the patients’ overall thrombotic risk profile. Information on possible venous compression by the primary tumor or metastatic lymph nodes was not systematically available, so its contribution to venous events could not be assessed. It should be acknowledged that current oncology guidelines recommend a cautious and restrictive use of ESA in patients with cancer due to their increased risk of thromboembolic events [53]. Regarding venous access devices, including central venous catheters, port-a-caths and peripherally inserted central catheters, it should be noted that the study population consisted exclusively of patients managed in an outpatient setting, which may partly explain the limited availability of these data. Complete-case laboratory analyses excluded 12.4% of patients because of data availability rather than clinical selection, although differences between patients with and without available results may have influenced the findings. Finally, the Khorana analysis included only four venous thromboembolism events and should therefore be interpreted cautiously.

## 5. Conclusions

In our cohort of patients with urological malignancies, advanced cancer stage was the most consistent factor associated with thrombosis. Thrombotic events occurred in 2.97% of patients, with arterial and venous events occurring in similar proportions, and were concentrated early after cancer diagnosis, with a median time to thrombosis of three months. Exploratory analyses suggested a possible relationship between pre-existing cardiovascular disease and arterial thrombosis, which requires confirmation in larger cohorts. The inflammatory indices did not distinguish patients with thrombosis or improve the clinical model. The Khorana score also showed no evidence of useful discrimination for venous thromboembolism among patients receiving systemic chemotherapy, although the small number of events precluded firm conclusions. Larger prospective studies are needed to confirm these findings.

## Figures and Tables

**Table 1:** Baseline demographic and oncological characteristics of the study population. Note: Percentages may not sum to 100% due to rounding.

Characteristic	Overall (Patients)	No Thrombosis *N* = 1013	Thrombosis *N* = 31	*p*-Value
**Median age**, years (IQR)	70.0 (64.0, 75.0)	69.0 (64.0, 75.0)	73.0 (66.0, 76.0)	0.2
**Sex**				0.2
Male, *n* (%)	892 (85.4%)	868 (85.7%)	24 (77.4%)	
Female, *n* (%)	152 (14.6%)	145 (14.3%)	7 (22.6%)	
**Cancer type**				0.029
Prostate, *n* (%)	440 (42.1%)	429 (42.3%)	11 (35.5%)	
Renal, *n* (%)	110 (10.5%)	104 (10.3%)	6 (19.4%)	
Bladder/renal pelvis/ureter, *n* (%)	479 (45.9%)	466 (46.0%)	13 (41.9%)	
Penile, *n* (%)	1 (0.1%)	0 (0.0%)	1 (3.2%)	
Testicular, *n* (%)	14 (1.3%)	14 (1.4%)	0 (0.0%)	
**Cancer stage at diagnosis**				0.027
Stage 0, *n* (%)	97 (9.3%)	96 (9.5%)	1 (3.2%)	
Stage I, *n* (%)	336 (32.2%)	330 (32.6%)	6 (19.4%)	
Stage II, *n* (%)	211 (20.2%)	207 (20.4%)	4 (12.9%)	
Stage III, *n* (%)	179 (17.1%)	173 (17.1%)	6 (19.4%)	
Stage IV, *n* (%)	221 (21.2%)	207 (20.4%)	14 (45.2%)	
**Histopathological grade**				0.5
Low, *n* (%)	115 (11.2%)	113 (11.4%)	2 (6.5%)	
Intermediate, *n* (%)	362 (35.3%)	353 (35.5%)	9 (29.0%)	
High, *n* (%)	548 (53.5%)	528 (53.1%)	20 (64.5%)	
Missing, *n* (%)	19	19	0	

**Table 2:** Characteristics of thrombotic events in the study cohort—ATE included myocardial infarction (MI), ischemic stroke, peripheral arterial events (including peripheral artery thrombosis, such as iliac artery thrombosis) and other arterial events (including splenic infarction). VTE included deep vein thrombosis (DVT) (including lower or upper extremity DVT), pulmonary embolism (PE) and thrombosis in other venous territories (including renal vein and inferior vena cava thrombosis). Tumor thrombi were not included as thrombotic events.

ID	Cancer Type	Stage at dx	Event Type	Location/Description	Symptomatic/Incidental
P06	Prostate	Stage IV	Arterial thrombosis	MI	Not documented
P21	Urothelial	Stage IV	Arterial thrombosis	Peripheral arterial event	Incidental
P25	Urothelial	Stage I	Arterial thrombosis	Peripheral arterial event	Incidental
P10	Prostate	Stage II	Arterial thrombosis	MI	Symptomatic
P26	Urothelial	Stage I	Arterial thrombosis	MI	Not documented
P28	Urothelial	Stage II	Arterial thrombosis	Peripheral arterial event	Symptomatic
P31	Urothelial	Stage IV	Arterial thrombosis	MI	Symptomatic
P01	Penile	Stage III	Arterial thrombosis	Other arterial events	Incidental
P07	Prostate	Stage IV	Arterial thrombosis	Stoke	Symptomatic
P18	Urothelial	Stage I	Arterial thrombosis	MI	Not documented
P27	Urothelial	Stage IV	Arterial thrombosis	MI	Symptomatic
P20	Prostate	Stage IV	Arterial thrombosis	Stroke	Symptomatic
P16	Urothelial	Stage I	Arterial thrombosis	Stroke	Symptomatic
P23	Urothelial	Stage 0	Arterial thrombosis	Peripheral arterial event	Incidental
P05	Prostate	Stage IV	Arterial thrombosis	Peripheral arterial event	Incidental
P03	Prostate	Stage IV	Arterial thrombosis	Stroke	Symptomatic
P02	Renal	Stage III	Arterial thrombosis	MI	Not documented
P13	Prostate	Stage II	Venous thrombosis	EP	Incidental
P14	Prostate	Stage IV	Venous thrombosis	DVT	Symptomatic
P30	Urothelial	Stage IV	Venous thrombosis	DVT	Symptomatic
P11	Renal	Stage III	Venous thrombosis	EP	Symptomatic
P19	Prostate	Stage III	Venous thrombosis	DVT	Symptomatic
P29	Renal	Stage II	Venous thrombosis	Other venous territories	Incidental
P08	Urothelial	Stage IV	Venous thrombosis	EP	Symptomatic
P22	Renal	Stage III	Venous thrombosis	Other venous territory	Incidental
P04	Renal	Stage IV	Venous thrombosis	Other venous territory	Incidental
P12	Urothelial	Stage I	Venous thrombosis	EP	Incidental
P24	Renal	Stage I	Venous thrombosis	DVT	Symptomatic
P17	Prostate	Stage III	Venous thrombosis	PE	Incidental
P15	Prostate	Stage IV	Venous thrombosis	DVT	Not documented
P09	Urothelial	Stage IV	Venous thrombosis	DVT	Symptomatic

**Table 3:** Primary and major sensitivity Firth models—Firth penalized logistic regression. The cancer-adjusted model also included cancer group; its full coefficients are reported in Supplementary Table S2. The purpose of sensitivity models was coefficient stability, not cancer-specific risk estimation.

Model	Predictor	aOR	95% CI	*p*-Value
Primary	Age, per 10 years	1.08	0.708–1.67	0.720
Primary	Cardiovascular disease	2.11	0.966–4.80	0.061
Primary	No. of CV risk factors	1.18	0.846–1.62	0.322
Primary	Stage, per one-level increase	1.60	1.21–2.17	<0.001
Cancer-adjusted sensitivity	Age, per 10 years	1.29	0.837–2.01	0.250
Cancer-adjusted sensitivity	Cardiovascular disease	2.04	0.913–4.71	0.083
Cancer-adjusted sensitivity	No. of CV risk factors	1.09	0.768–1.51	0.630
Cancer-adjusted sensitivity	Stage, per one-level increase	1.89	1.38–2.61	<0.001
Stage IV sensitivity	Stage IV vs. stages 0–III	3.27	1.57–6.74	0.002
Stage IV+ cancer sensitivity	Stage IV vs. stages 0–III	4.18	1.87–9.29	<0.001

**Table 4:** Inflammatory indices: individual and incremental discrimination—Complete-case population *n* = 915 with 26 thrombotic events. Paired DeLong tests compare each expanded model with the clinical model; BH adjustment applied across the four incremental comparisons.

Analyses	AUC	95% CI	DeLong *p*-Value vs. Clinical Model	BHq
PLR alone	0.513	0.395–0.632	Not applicable	Not applicable
NLR alone	0.509	0.390–0.628	Not applicable	Not applicable
SII alone	0.501	0.374–0.628	Not applicable	Not applicable
SIRI alone	0.487	0.367–0.608	Not applicable	Not applicable
Clinical model (complete-case)	0.757	0.683–0.831	Reference	-
Clinical + NLR	0.758	0.684–0.832	0.781	0.963
Clinical + PLR	0.760	0.684–0.835	0.487	0.963
Clinical + SIRI	0.757	0.682–0.832	0.963	0.963
Clinical + SII	0.760	0.685–0.835	0.568	0.963

## Data Availability

The raw data supporting the conclusions of this article will be made available by the authors on request.

## Supplementary Materials

The following supporting information can be downloaded at: https://www.mdpi.com/article/10.3390/diagnostics16203298/s1, Table S1. Diagnostic cancer stage by thrombotic event type. Table S2. Full coefficients from the cancer-adjusted sensitivity model. Table S3. Recorded treatment exposure by thrombosis status. Table S4. Univariable Firth regression comparisons of recorded treatment exposure and thrombosis, with Benjamini–Hochberg-adjusted q-values.

## Abbreviations

The following abbreviations are used in this manuscript:

CAT	Cancer-associated thrombosis
VTE	Venous thrombotic event
ATE	Arterial thrombotic event
GnRH	Gonadotropin-releasing hormone
PARP	Poly (ADP-ribose) polymerase
DVT	Deep vein thrombosis
PE	Pulmonary embolism
CT	Computed tomography
TNM	Tumor–Node–Metastasis
ISUP	International Society of Urological Pathology
WHO	World Health Organization
BCG	Bacillus Calmette–Guérin
BMI	Body Mass Index
ASCO	American Society of Clinical Oncology
NLR	Neutrophil-to-lymphocyte ratio
PLR	Platelet-to-lymphocyte ratio
SIRI	Systemic inflammation response index
SII	Systemic immune-inflammation index
IQR	Interquartile range
OR	Odds ratio
CI	Confidence intervals
ROC	Receiver Operating Characteristic
AUC	Area under the curve
PPV	Positive predictive value
NPV	Negative predictive value
BH	Benjamini-Hochberg
ICI	Immune checkpoint inhibitors
ADT	Androgen deprivation therapy
ESA	Erythropoiesis-stimulating agents
