# Supplementary material for: Arterial and Venous Thrombotic Events in Urological Malignancy—A Real-World Cohort Study from a Tertiary Urology Referral Center
Source: Diagnostics (Basel). 2026 Oct 11;16(20):3298. doi: 10.3390/diagnostics16203298 (PMC13703362; doi:10.3390/diagnostics16203298)
Supplement: Supplementary file 1 [file diagnostics-16-03298-s001.zip › diagnostics-4564669-supplementary.pdf]

Supplementary file

Supplementary Table S1. Diagnostic cancer stage by thrombotic event type.

Cancer stage	Patients with no thrombosis (n=1,013)	ATE (n=17)	VTE (n=14)
Stage 0	96 (9.5%)	1 (5.9%)	0 (0.0%)
Stage I	330 (32.6%)	4 (23.5%)	2 (14.3%)
Stage II	207 (20.4%)	2 (11.8%)	2 (14.3%)
Stage III	173 (17.1%)	2 (11.8%)	4 (28.6%)
Stage IV	207 (20.4%)	8 (47.1%)	6 (42.9%)

Values are n (%); percentages are calculated within each column; n=number of patients. ATE, arterial thrombotic events; VTE, venous thromboembolic events.

Supplementary Table S2. Full coefficients from the cancer-adjusted sensitivity model

Predictor	aOR	95% CI	p
Age, per 10 years	1.29	0.837-2.01	0.250
Cardiovascular disease	2.04	0.913-4.71	0.083
No. of CV risk factors	1.09	0.768-1.51	0.630
Stage, per one-level increase	1.89	1.38-2.61	<0.001
Renal vs prostate	3.66	1.18-10.6	0.025
Urothelial vs prostate	2.78	1.12-6.92	0.028
Other vs prostate	16.3	1.45-111	0.028

Prostate cancer was the reference category. 'Other' combines penile and testicular cancers for this sensitivity analysis only. Cancer coefficients are sparse nuisance adjustment parameters and should not be interpreted as definitive cancer-specific risk estimates.

Supplementary Table S3. Recorded treatment exposure by thrombosis status

Treatment	Overall (patients)	No thrombosis, n = 1,013	Thrombosis, n = 31	p-value
Surgery	665 (63.7%)	646 (63.8%)	19 (61.3%)	0.9
Chemotherapy	79 (7.6%)	70 (6.9%)	9 (29.0%)	<0.001
Hormone therapy	299 (28.6%)	289 (28.5%)	10 (32.3%)	0.7
Intravesical BCG	127 (12.2%)	126 (12.4%)	1 (3.2%)	0.2
Intravesical chemotherapy	174 (16.7%)	170 (16.8%)	4 (12.9%)	0.8
Any intravesical therapy	299 (28.6%)	294 (29.0%)	5 (16.1%)	0.2
Radiotherapy	111 (10.6%)	106 (10.5%)	5 (16.1%)	0.4
Immunotherapy	25 (2.4%)	21 (2.1%)	4 (12.9%)	0.005
Targeted therapy	10 (1.0%)	10 (1.0%)	0 (0.0%)	>0.9

These are recorded exposure variables, not necessarily therapies active at the thrombotic event. Unadjusted comparisons are exploratory and susceptible to confounding by indication and temporal ambiguity. n=number of patients.

Supplementary Table S4. Univariable Firth regression comparisons of recorded treatment exposure and thrombosis, with Benjamini–Hochberg-adjusted q-values.

Recorded treatment exposure	Patients exposed, n	Thrombotic events among exposed, n	Firth p	BH q
Systemic chemotherapy	79	9	<0.001 (0.00016)	0.001
Immunotherapy	25	4	0.002	0.011
Any intravesical therapy	299	5	0.125	0.309
Intravesical BCG	127	1	0.137	0.309
Radiotherapy	111	5	0.262	0.472
Hormone therapy	299	10	0.604	0.788
Intravesical chemotherapy	174	4	0.675	0.788
Surgery	665	19	0.745	0.788
Targeted therapy	10	0	0.788	0.788

Each exposure was evaluated in a separate univariable Firth penalized logistic regression model. Benjamini–Hochberg adjustment was applied across the nine comparisons shown. No thrombotic events occurred among the 10 patients with recorded targeted therapy exposure; this result is included for completeness and offers limited information about an association. n=number of patients.
